# Supplementary material for: Harnessing a catalytic lysine residue for the one-step preparation of homogeneous antibody-drug conjugates
Source: Nat Commun. 2017 Oct 24;8:1112. doi: 10.1038/s41467-017-01257-1 (PMC5653646; doi:10.1038/s41467-017-01257-1)
Supplement: Supplementary file 1 — Supplementary Information [file 41467_2017_1257_MOESM1_ESM.pdf]

Supplementary Figures

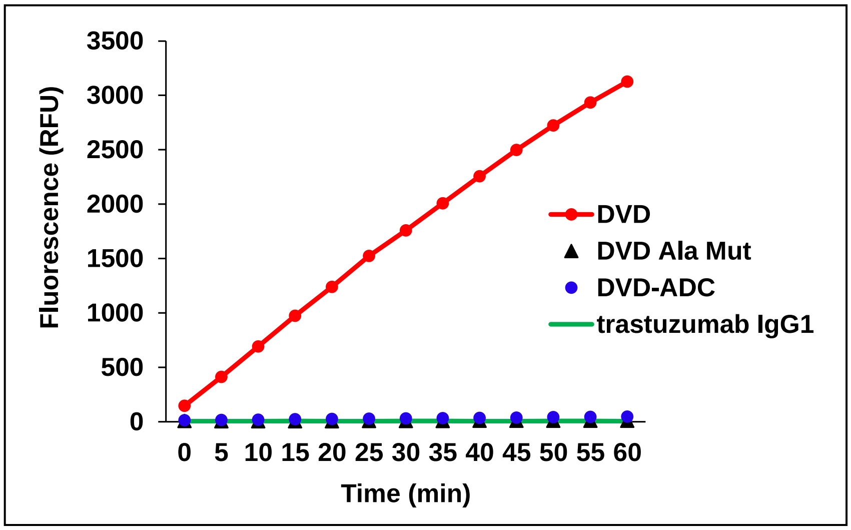

Supplementary Figure 1. Catalytic assay indicates complete and specific conjugation to defined Lys. The catalytic activity of the reactive Lys of h38C2 contained in the DVDs was measured directly using methodol as a substrate, which is converted to a fluorescent aldehyde via a retro-aldol reaction and detected as described in Fig. 1. The assembled DVD-ADC (blue) was catalytically inactive due to amide formation at the reactive Lys, indicating specific and complete conjugation. Unconjugated anti-HER2 DVD (red) was used as a positive control and anti-HER2 DVD Ala Mut (where the reactive Lys of h38C2 was mutated to Ala, black) and trastuzumab IgG1 (green) as negative controls (mean ± s.d. of triplicates).

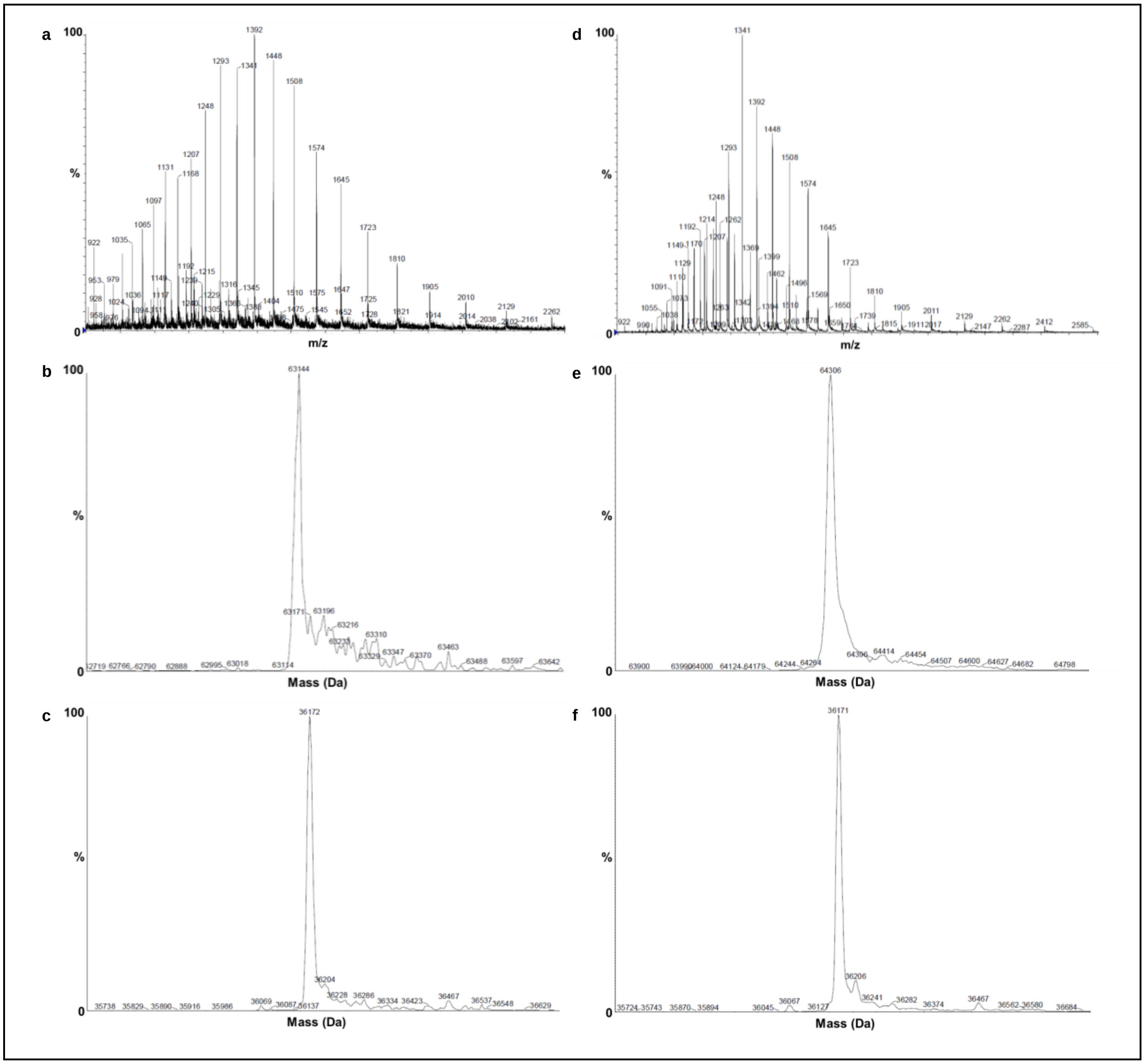

Supplementary Figure 2. Non-deconvoluted and zoomed in deconvoluted ESI-MS spectra of anti-HER2 DVD and anti-HER2 DVD-ADC corresponding to Figure 2c. (**a**) Non-deconvoluted spectrum of anti-HER2 DVD. (**b**) Zoomed in deconvoluted spectrum of anti-HER2 DVD heavy chain. (**c**) Zoomed in deconvoluted spectrum of anti-HER2 DVD light chain. (**d**) Non-deconvoluted spectrum of anti-HER2 DVD-ADC. (**e**) Zoomed in deconvoluted spectrum of anti-HER2 DVD-ADC heavy chain. (**f**) Zoomed in deconvoluted spectrum of anti-HER2 DVD-ADC light chain.

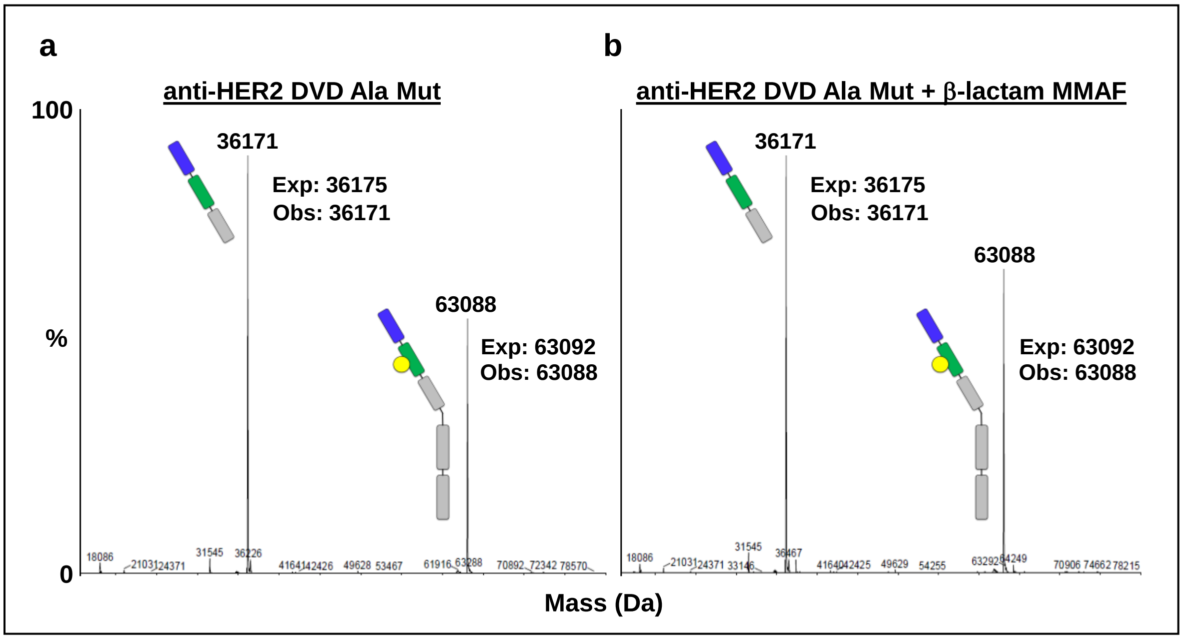

Supplementary Figure 3. ESI-MS of anti-HER2 DVD Ala Mut incubated with β-lactam MMAF shows no detectable conjugation. (**a**) ESI-MS of reduced (10 mM DTT) and deglycosylated (PNGase F) anti-HER2 DVD Ala Mut with expected and observed masses of the light and heavy chain. (**b**) ESI-MS of reduced (10 mM DTT) and deglycosylated (PNGase F) anti-HER2 DVD Ala Mut incubated with β-lactam MMAF reveals no conjugation on the heavy or light chain.

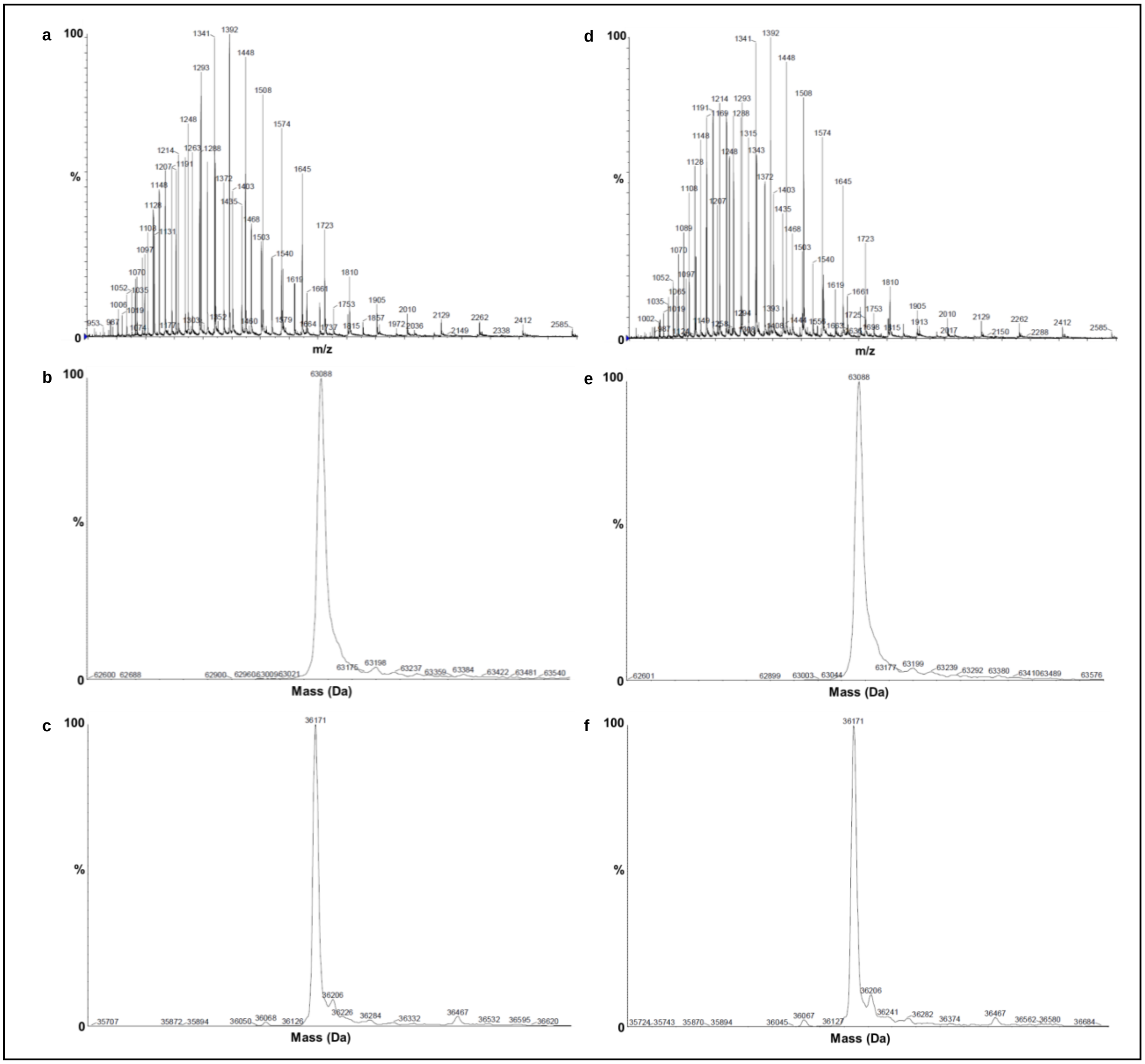

Supplementary Figure 4. Non-deconvoluted and zoomed in deconvoluted ESI-MS spectra of anti-HER2 DVD Ala Mut and anti-HER2 DVD Ala Mut incubated with β–lactam MMAF corresponding to Supplementary Figure 3. (**a**) Non-deconvoluted spectrum of anti-HER2 DVD Ala Mut. (**b**) Zoomed in deconvoluted spectrum of anti-HER2 DVD Ala Mut heavy chain. (**c**) Zoomed in deconvoluted spectrum of anti-HER2 DVD Ala Mut light chain. (**d**) Non-deconvoluted spectrum of anti-HER2 DVD Ala Mut incubated with β-lactam MMAF. (**e**) Zoomed in deconvoluted spectrum of the heavy chain of anti-HER2 DVD Ala Mut incubated with β-lactam MMAF. (**f**) Zoomed in deconvoluted spectrum of the light chain of anti-HER2 DVD Ala Mut incubated with β-lactam MMAF.

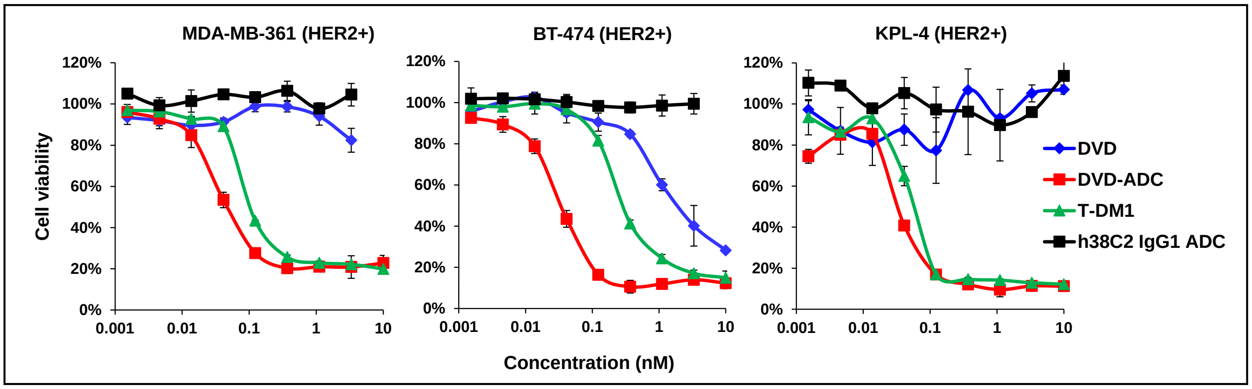

Supplementary Figure 5. Anti-HER2 DVD-ADC is highly potent against several HER2-expressing BC cell lines. Cytotoxicity of anti-HER2 DVD-ADC (red) following incubation with HER2+ BC cell lines (MDA-MB-361, BT-474, and KPL-4) for 72 h at 37ºC (mean ± s.d. of triplicates). Unconjugated anti-HER2 DVD (blue) was less toxic in all HER2-expressing cell lines. T-DM1 (green) was used as a positive control and h38C2 IgG1 ADC (black; h38C2 IgG1 conjugated to β-lactam MMAF) as a negative control.

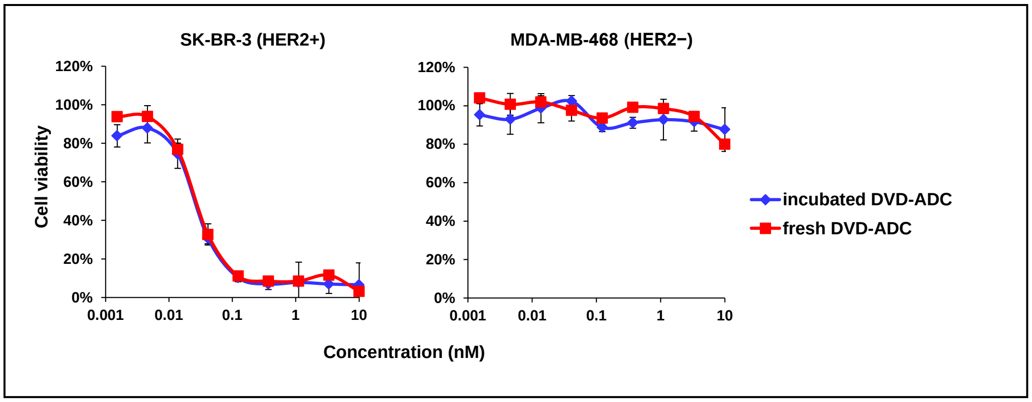

Supplementary Figure 6. Anti-HER2 DVD-ADC is highly stable in human plasma. Anti-HER2 DVD-ADC was incubated in human plasma for 72 h at 37ºC, diluted to the indicated concentrations with PBS, and then incubated with HER2+ BC cell line SK-BR-3 and HER2− BC cell line MDA-MB-468 for 72 h at 37ºC (mean ± s.d. of triplicates). Incubated anti-HER2 DVD-ADC (blue) did not have significantly different activity ($p = 0.3633$; extra sum-of-squares F-test) when compared to fresh anti-HER2 DVD-ADC (red), indicating no drug loss and high stability at the drug attachment site.

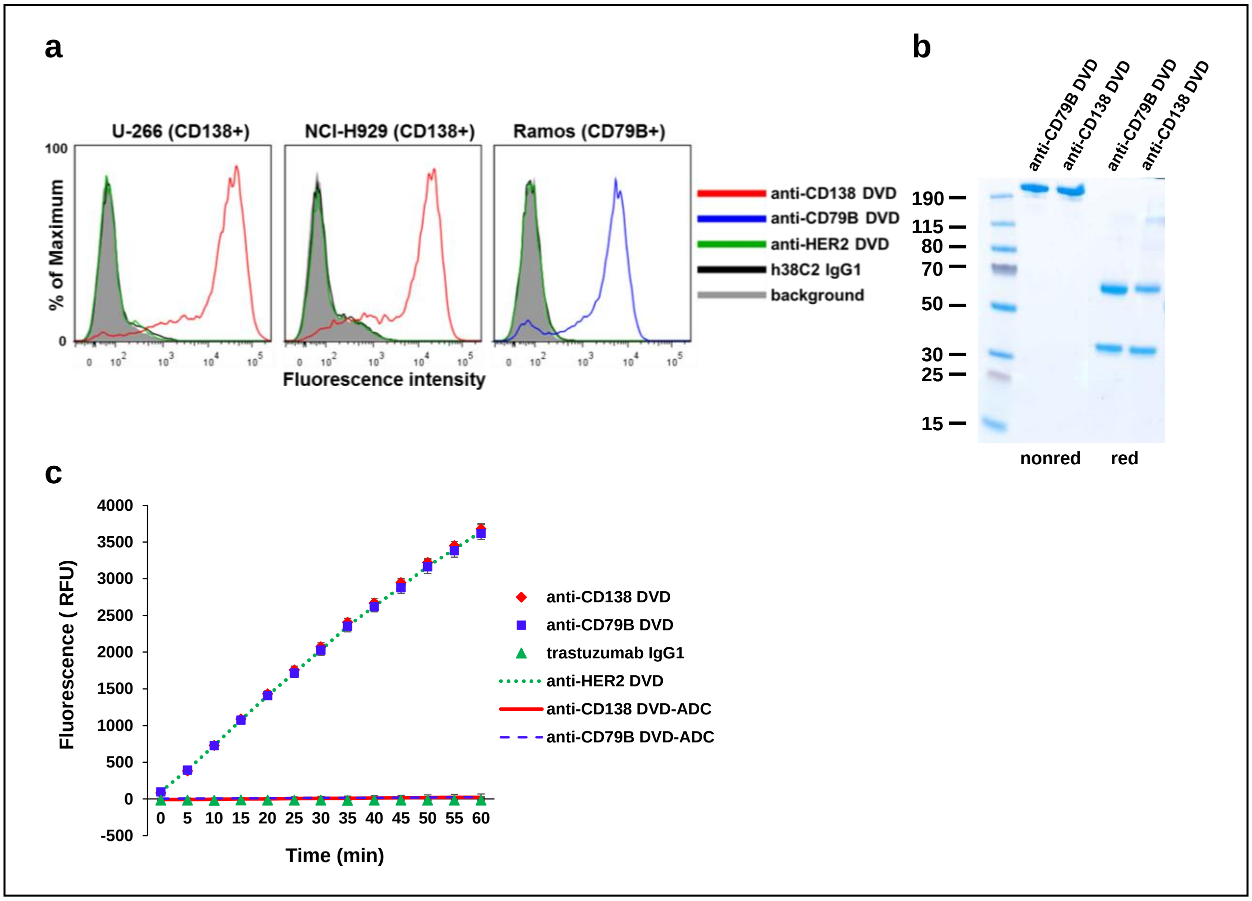

Supplementary Figure 7. The modularity of the DVD-ADC platform is shown by switching from HER2 to CD138 and CD79B targeting. (**a**) Flow cytometry showing specific binding of anti-CD138 DVD (red) and anti-CD79B DVD (blue) to CD138-expressing multiple myeloma cell lines (U-266 and NCI-H929) and CD79B-expressing Burkitt lymphoma cell line (Ramos), respectively. (Please see Methods for a cautionary note regarding the NCI-H929 cell line). Anti-HER2 DVD (green) and h38C2 IgG1 (black) were used as negative controls. (**b**) Coomassie stained SDS-PAGE confirmed the purity of both anti-CD138 DVD and anti-CD79B DVD under non-reducing (expected ~200 kDa) and reducing conditions (expected heavy chain ~63 kDa, light chain ~36 kDa). Molecular weights from a pre-stained protein ladder are shown on the left. (**c**) The catalytic activity of the reactive Lys of h38C2 contained in the DVDs was measured directly as described in Fig. 1. Unconjugated anti-CD138 DVD (red diamonds), anti-CD79B DVD (blue squares), and anti-HER2 DVD (green dotted line) all had identical catalytic activity (mean ± s.d. of triplicates). This was confirmed by determining that the slope of the anti-HER2 DVD was not significantly different from the slope of the anti-CD138 DVD ($p = 0.2809$) or the anti-CD79B DVD ($p = 0.7539$) using linear regression analysis. Anti-CD138 DVD-ADC (red line) and anti-CD79B DVD-ADC (blue dashed line) were both catalytically inactive, indicating specific and complete conjugation at the reactive Lys. Trastuzumab IgG1 (green triangles) was used as a negative control.

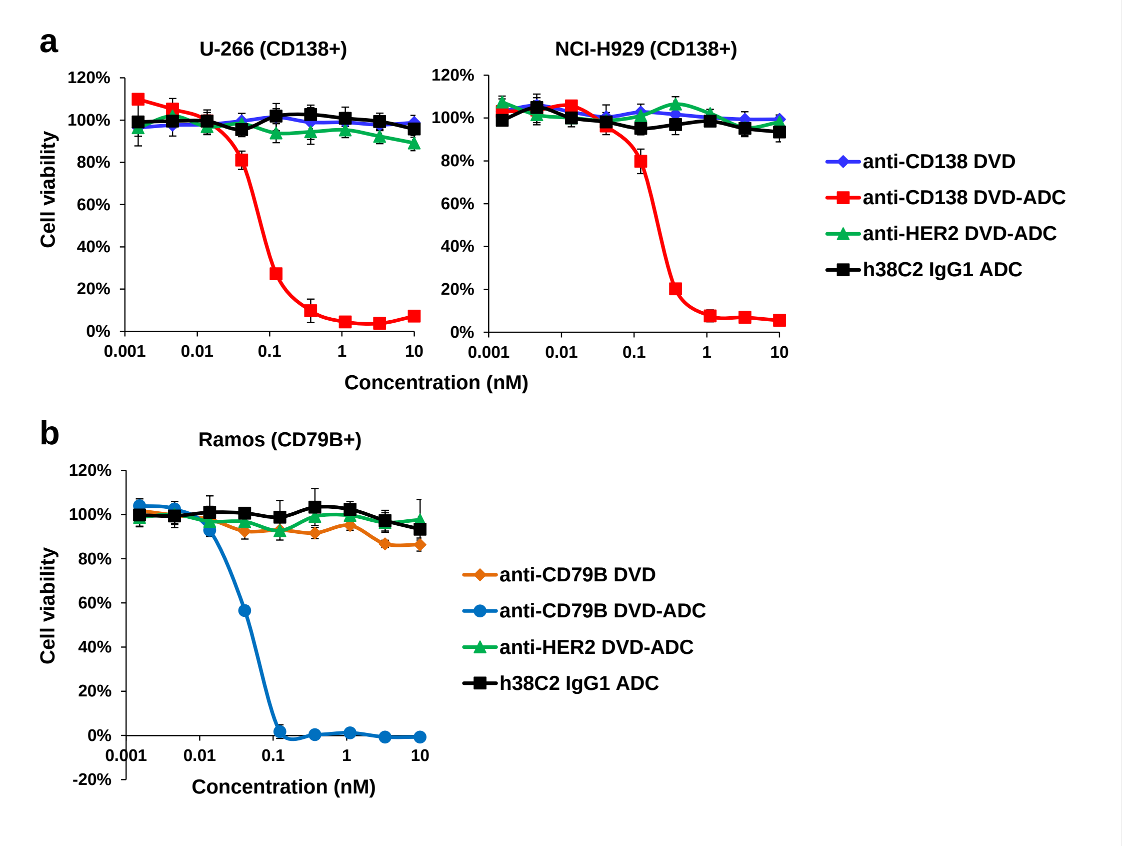

Supplementary Figure 8. Anti-CD138 and anti-CD79B DVD-ADCs are highly potent against target-expressing cancer cell lines. (**a**) Cytotoxicity of anti-CD138 DVD-ADC conjugate (red) following incubation with CD138+ MM cell lines U-266 and NCI-H929 for 72 h at 37ºC (mean ± s.d. of triplicates). (Please see Methods for a cautionary note regarding the NCI-H929 cell line). (**b**) Cytotoxicity of anti-CD79B DVD-ADC (blue) following incubation with CD79B+ Burkitt lymphoma cell line Ramos for 72 h at 37ºC (mean ± s.d. of triplicates). Unconjugated anti-CD138 DVD (blue), unconjugated anti-CD79B DVD (orange), anti-HER2 DVD-ADC (green), and h38C2 IgG1 ADC (black) were used as negative controls.

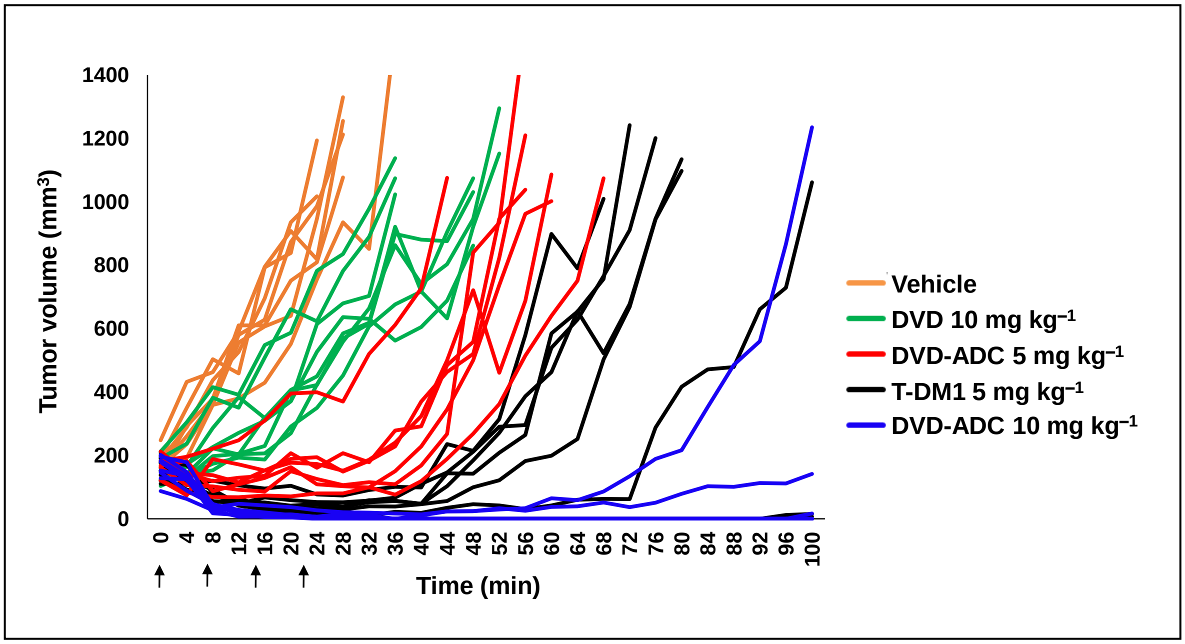

Supplementary Figure 9. Anti-HER2 DVD-ADC is highly potent *in vivo*. Human BC cell line KPL-4 was xenografted into the mammary fat pads of female NSG mice, grown to ~150 mm^3, randomized into 5 groups comprising 7 or 8 mice each, and treated with i.v. (tail vein) injections of the indicated ADCs and controls once a week for 4 weeks. The benchmark ADC, T-DM1 (black), was used as a positive control. Each line represents an individual animal. At the end of 100 days, the DVD-ADC at 10 mg kg^{-1} (blue) outperformed T-DM1 (black) with 5/8 cures and 7/8 survivals as opposed to 0/8 cures and 2/8 survivals, respectively.

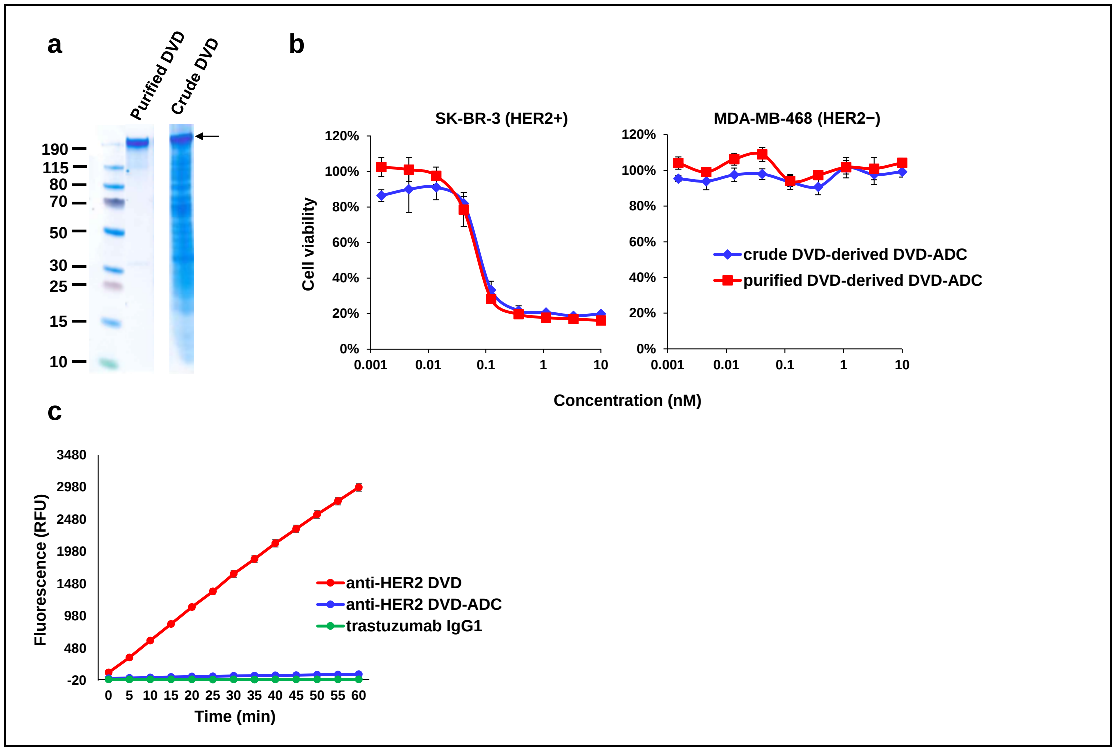

Supplementary Figure 10. Anti-HER2 DVD-ADC can be assembled from crude DVD. (**a**) Coomassie stained SDS-PAGE of purified anti-HER2 DVD prepared using Protein A compared to crude anti-HER2 DVD prepared using ammonium sulfate precipitation and dialysis. The desired band is indicated with an arrow (expected ~200 kDa). Molecular weights from a pre-stained protein ladder are shown on the left. (**b**) Cytotoxicity of anti-HER2 DVD-ADCs prepared using purified anti-HER2 DVD (red) versus crude anti-HER2 DVD (blue) following incubation with HER2+ BC cell line SK-BR-3 and HER2− BC cell line MDA-MB-468 for 72 h at 37ºC (mean ± s.d. of triplicates). Using crude DVD did not diminish the potency of the resulting DVD-ADC and there was no significant difference between the IC_{50} values ($p = 0.4089$; extra sum-of-squares F-test). (**c**) The catalytic activity of the reactive Lys of h38C2 contained in the DVDs was measured directly as described in Fig. 1 (mean ± s.d. of triplicates). Anti-HER2 DVD-ADC prepared using crude DVD was catalytically inactive, indicating specific and complete conjugation at the reactive Lys despite the presence of additional proteins in the crude reaction. Unconjugated anti-HER2 DVD (red) was used as a positive control and trastuzumab IgG1 (green) as a negative control.

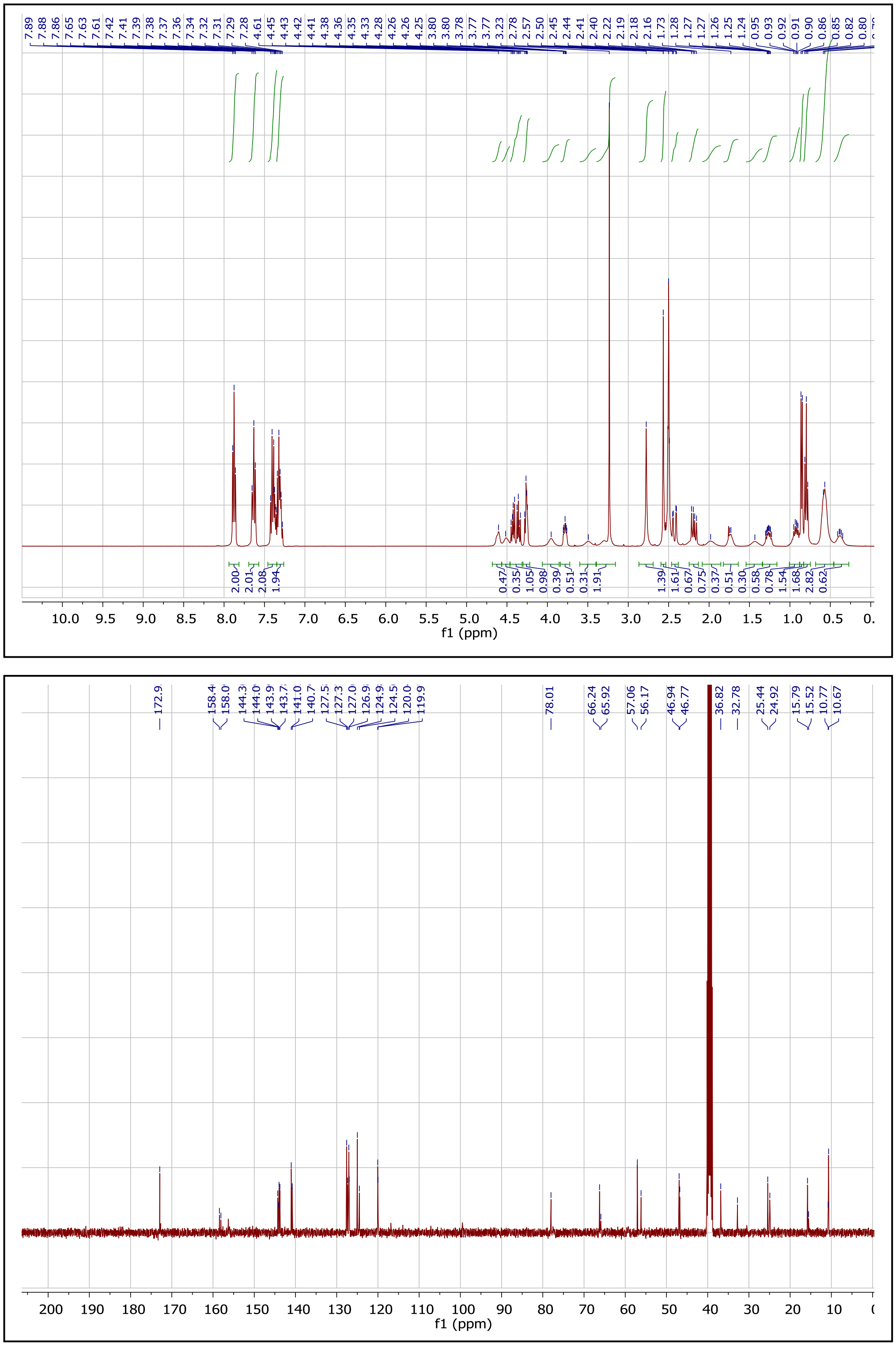

Supplementary Figure 11. NMR spectra for Fmoc-dolaisoleucine. ^{1}H (top) and ^{13}C (bottom) NMR spectra for (3R,4S,5S)-4-((((9H-fluoren-9-yl)methoxy)carbonyl)(methyl)amino)-3-methoxy-5-methylheptanoic acid (Fmoc-dolaisoleucine, **3**).

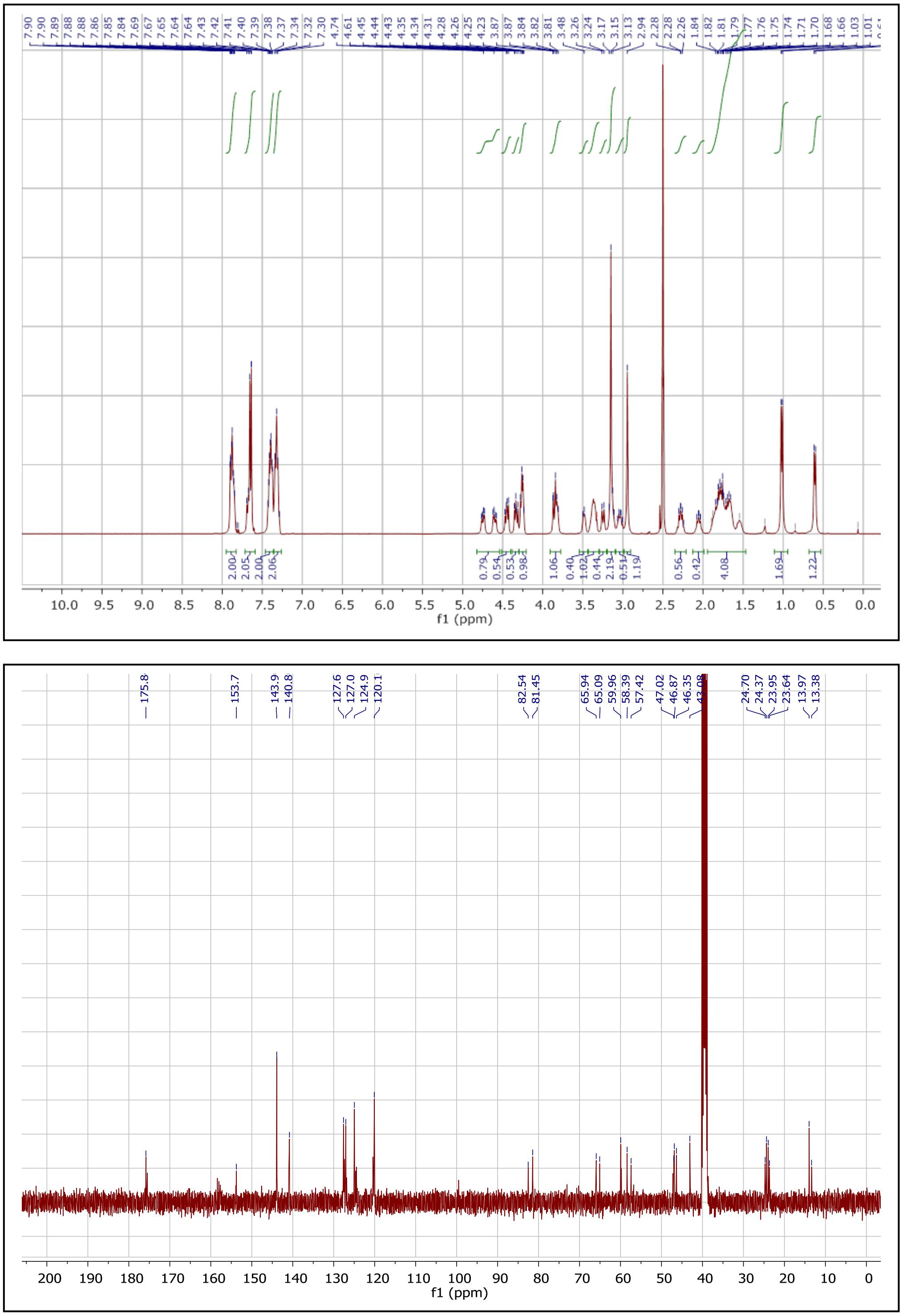

Supplementary Figure 12. NMR spectra for Fmoc-dolaproline. ^{1}H (top) and ^{13}C (bottom) NMR spectra for (2R,3R)-3-((S)-1-(((9H-fluoren-9-yl)methoxy)carbonyl)pyrrolidin-2-yl)-3-methoxy-2-methylpropanoic acid (Fmoc-dolaproline, **6**).

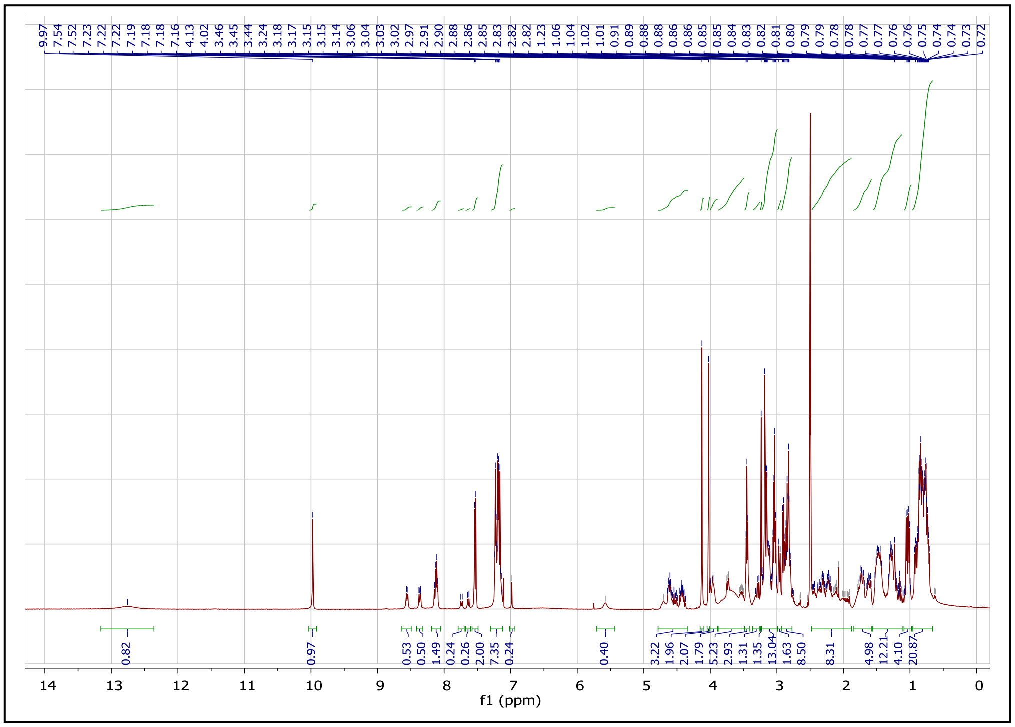

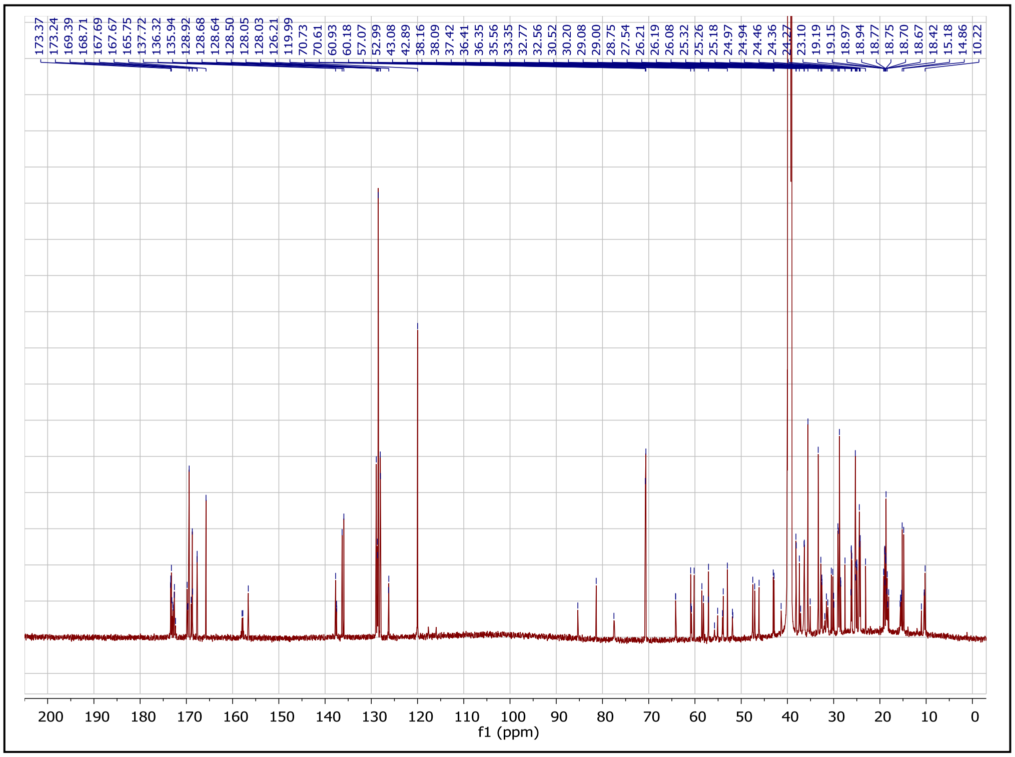

Supplementary Figure 13. NMR spectra for β-lactam MMAF. ^{1}H (top) and ^{13}C (bottom) NMR spectra for ((2R,3R)-3-((S)-1-((3R,4S,5R)-4-((S)-N,3-dimethyl-2-((S)-3-methyl-2-(N-methyl-6-(2-(2-oxo-2-((4-(3-oxo-3-(2-oxoazetidin-1-yl)propyl)phenyl)amino)ethoxy)acetamido)hexanamido)butanamido)butanamido)-3-methoxy-5-methylheptanoyl)pyrrolidin-2-yl)-3-methoxy-2-methylpropanoyl)-L-phenylalanine (β-lactam MMAF, **14**).

Supplementary Tables

Supplementary Table 1. ***In silico*** **T-cell epitope analysis of the anti-HER2 DVD.**

Protein Component	Length	Assessments	EpiMatrix Hits	EpiMatrix Score (Raw)	Tregitope-Adjusted EpiMatrix Score*
Light Chain	332	2592	168	21.67	-17.44
Heavy Chain	574	4528	255	2.53	-41.93
Whole Antibody	457	3592	238	35.2	-34.29
trastuzumab VL	107	792	54	28.48	-46.91
h38C2 VL	112	832	69	58.82	21.87
CL	107	792	35	-22.29	-36.1
trastuzumab VH	120	896	42	-8.77	-83.92
h38C2 VH	118	880	73	63.69	-25.48
CH1-hinge-CH2-CH3	330	2576	130	-13.38	-34.92

*Green values indicate a low, red values a high risk of immunogenicity.

Supplementary Table 2. ***In silico*** **T-cell epitope analysis of the junctional regions connecting outer and inner Fv of the anti-HER2 DVD.**

Protein Component	Spacer Sequence	EpiMatrix Hits	EpiMatrix Cluster Score*
Light Chain	QGTKVEIK**ASTKGP**ELQMTQSP	3	-4.85
Heavy Chain	GTLVTVSS**ASTKGP**EVQLVESG	5	-2.88

*Black values indicate a neutral risk of immunogenicity.

Supplementary Table 3. *In silico* T-cell epitope analysis of the Lys (K225) conjugation point in h38C2 VH.

Input Sequence	Cluster Sequence	EpiMatrix Hits	EpiMatrix Cluster Score*
K225K	EDTGIYYC**K**TYFYSFSY	0	-7.42
K225A	EDTGIYYC**A**TYFYSFSY	0	-7.42
K225C	EDTGIYYC**C**TYFYSFSY	0	-7.42
K225D	EDTGIYYC**D**TYFYSFSY	0	-7.42
K225E	EDTGIYYC**E**TYFYSFSY	0	-7.42
K225F	EDTGIYYC**F**TYFYSFSY	4	0.69
K225G	EDTGIYYC**G**TYFYSFSY	0	-7.42
K225H	EDTGIYYC**H**TYFYSFSY	0	-7.42
K225I	EDTGIYYC**I**TYFYSFSY	2	-2.90
K225L	EDTGIYYC**L**TYFYSFSY	3	-1.14
K225M	EDTGIYYC**M**TYFYSFSY	3	-1.23
K225N	EDTGIYYC**N**TYFYSFSY	0	-7.42
K225P	EDTGIYYC**P**TYFYSFSY	0	-7.42
K225Q	EDTGIYYC**Q**TYFYSFSY	0	-7.42
K225R	EDTGIYYC**R**TYFYSFSY	1	-5.57
K225S	EDTGIYYC**S**TYFYSFSY	0	-7.42
K225T	EDTGIYYC**T**TYFYSFSY	0	-7.42
K225V	EDTGIYYC**V**TYFYSFSY	2	-3.10
K225W	EDTGIYYC**W**TYFYSFSY	2	-3.83
K225Y	EDTGIYYC**Y**TYFYSFSY	3	-1.49

*Black values indicate a neutral risk of immunogenicity.

Supplementary Methods

Synthesis of β-lactam-MMAF

1) Synthesis of Fmoc-dolaisoleucine and Fmoc-dolaproline

General information:

Dimethylformamide (DMF) and dichloromethane (CH_2Cl_2) were purified by passing through a solvent column of activated alumina (A-1). Unless otherwise indicated, all starting materials were purchased from various commercial sources and were used without further purification. Reactions were conducted under an atmosphere of argon using flamed-dried glassware. Standard techniques for handling air-sensitive compounds were employed for all the operations. Removal of solvents was accomplished on a rotary evaporator at reduced pressure.

Physical properties and spectroscopic measurements:

^{1}H NMR spectra were recorded on a Bruker spectrometer at 400 MHz. ^{13}C NMR spectra were recorded on a 400 MHz Bruker spectrometer at 100 MHz or on a 700 MHz Bruker spectrometer at 175 MHz for compound **14**. The proton signal for non-deuterated solvent (δ2.50 for DMSO) was used as an internal reference for ^{1}H NMR spectra. For ^{13}C NMR spectra, chemical shifts were reported relative to the δ39.5 resonance of DMSO. High-resolution mass spectra were recorded at the University of Illinois Mass Spectrometry Laboratory. Analytical thin layer chromatography (TLC) was performed on Kieselgel 60 F254 glass plates precoated with a 0.25-

mm thickness of silica gel. TLC plates were visualized with UV light and/or by staining with I_2 vapors. Column chromatography was performed using a Biotage Isolera purification system with Biotage pre-packed columns. LC-MS was performed on an Agilent Infinity LC with an Agilent Technologies 500 MS.

Synthesis of (3R,4S,5S)-3-Methoxy-5-methyl-4-(methylamino)heptanoic acid hydrochloride (dolaisoleucine hydrochloride, **2***):*

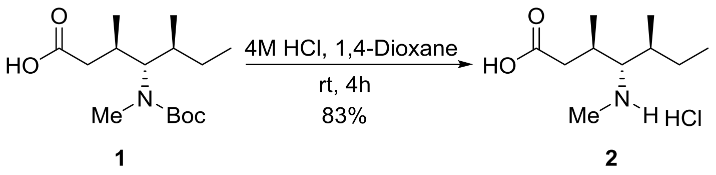

To Boc-dolaisoleucine **1**[1] (398 mg, 1.3 mmol, 1.0 eq) was added 1,4-dioxane (1.6 mL) and the solution was placed in an ice bath. 4 M HCl in 1,4-dioxane (3.3 mL, 13.1 mmol, 10 eq) was added dropwise and the resulting colorless solution was stirred at rt (4 h). The reaction mixture was concentrated under reduced pressure to give 248 mg of **2** as a beige solid (83%). The crude product was used in the next step without further purification. HRMS (ESI) calculated *m/z* for $C_{10}H_{22}NO_3$ $(M+H)^+$ 204.1600, found 204.1603.

Synthesis of (3R,4S,5S)-4-((((9H-fluoren-9-yl)methoxy)carbonyl)(methyl)amino)-3-methoxy-5-methylheptanoic acid (Fmoc-dolaisoleucine, **3***):*

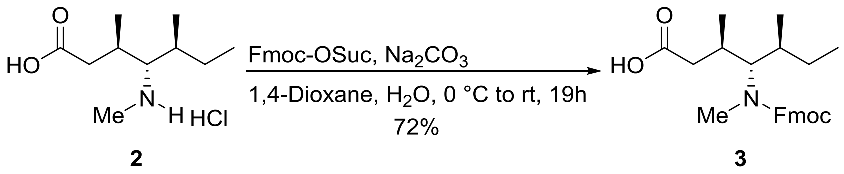

Dolaisoleucine•HCl **2** (248 mg, 1.0 mmol, 1.0 eq) was dissolved in H_2O (7.4 mL) and Na_2CO_3 (164 mg, 1.6 mmol, 1.5 eq) was added. The resulting solution was stirred in an ice bath for 20 min. A solution of Fmoc-succinimide (Fmoc-OSu; 523 mg, 1.6 mmol, 1.5 eq) in 1,4-dioxane (7.4 mL) was added to the solution in the ice bath. The resulting white suspension was stirred at rt overnight (19 h) and then diluted with H_2O (25 mL) and brine (10 mL). The mixture was extracted with EtOAc (2 x 25 mL), and the organic layer was washed with brine, dried ($MgSO_4$ and Na_2SO_4), filtered, and concentrated under reduced pressure. The crude product was purified by flash chromatography using a Biotage purification system with an increasing concentration of EtOAc in hexanes, then with an increasing concentration of 9:1:0.1 CH_2Cl_2:MeOH:AcOH in CH_2Cl_2 (up to 100%) to give 293 mg of **3** as a yellow solid (72%). The compound is a mixture of rotamers. ^{1}H NMR (400 MHz, DMSO-d_6) δ 7.88 (t, J = 7.0 Hz, 2.00H), 7.63 (t, J = 8.2 Hz, 2.01H), 7.46 – 7.35 (m, 2.08H), 7.35 – 7.26 (m, 1.94H), 4.61 (br s, 0.47H), 4.52 (br s, 0.35H), 4.39 (ddd, J = 28.9, 10.6, 6.5 Hz, 1.05H), 4.30 – 4.22 (m, 0.98H), 3.95 (br s, 0.39H), 3.78 (td, J = 7.4, 6.1, 2.8 Hz, 0.51H), 3.49 (br s, 0.31H), 3.23 (br s, 1.91H), 2.78 (s, 1.39H), 2.57 (s, 1.61H), 2.43 (dd, J = 15.8, 2.9 Hz, 0.67H), 2.19 (dd, J = 15.8, 9.0 Hz, 0.75H), 1.98 (br s, 0.37H), 1.74 (d, J = 6.1 Hz, 0.51H), 1.43 (br s, 0.30H), 1.27 (ddt, J = 16.1, 8.6, 4.4 Hz, 0.58H), 1.02 – 0.89 (m, 0.78H), 0.85 (d, J = 6.7 Hz, 1.54H), 0.80 (t, J = 7.3 Hz, 1.68H), 0.69 – 0.46 (m, 2.82H), 0.45 – 0.27 (m, 0.62H); ^{13}C NMR (100 MHz, DMSO) δ 172.93, 158.46, 158.09, 144.30, 144.09, 143.99, 143.78, 141.03, 140.79, 127.58, 127.37, 127.06, 126.98, 124.98, 124.50, 120.04, 119.97, 78.01, 66.24, 65.92, 57.06, 56.17, 46.94, 46.77, 36.82, 32.78, 25.44, 24.92, 15.79, 15.52, 10.77, 10.67. See Supplementary Fig. 11 for ^{1}H and ^{13}C NMR spectra. HRMS (ESI) calculated m/z for $C_{25}H_{32}NO_5$ $(M+H)^+$ 426.2280, found 426.2274.

*Synthesis of (2R,3R)-3-((S)-1-(((9H-fluoren-9-yl)methoxy)carbonyl)pyrrolidin-2-yl)-3-methoxy-2-methylpropanoic acid (Fmoc-dolaproline, **6**):*

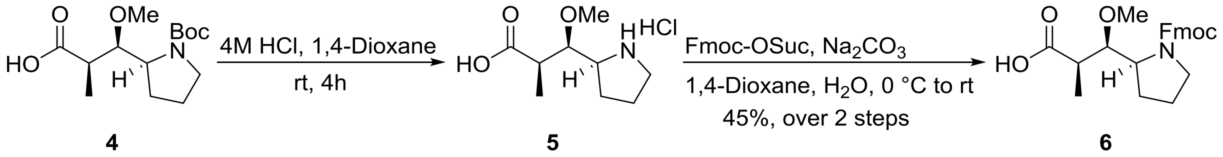

A solution of Boc-dolaproline **4**[2] (196 mg, 0.68 mmol, 1.0 eq) in 1,4-dioxane (0.82 mL) was placed in an ice bath. 4 M HCl in 1,4-dioxane (1.7 mL, 6.6 mmol, 10 eq) was added dropwise and the resulting colorless solution was stirred at rt for 4 h. The reaction mixture was concentrated under reduced pressure to give crude dolaproline•HCl (**5**) as a yellow gel. This was suspended in H_2O (4.9 mL) and Na_2CO_3 (108 mg, 1.0 mmol, 1.5 eq) was added. The resulting solution was stirred in an ice bath, then a solution of Fmoc-OSuc (345 mg, 1.0 mmol, 1.5 eq) in 1,4-dioxane (4.9 mL) was added. The resulting white suspension was allowed to warm to rt overnight (15 h), and diluted with H_2O (25 mL) and (10 mL brine). The reaction was extracted with EtOAc (2 x 25 mL), washed with brine, dried ($MgSO_4$ and Na_2SO_4), filtered, and concentrated under reduced pressure. The crude product was purified by flash chromatography using a Biotage purification system with an increasing concentration of EtOAc in hexanes, then with an increasing concentration of MeOH in CH_2Cl_2 (up to 10%) to give **6** as a pale yellow gel (126 mg, 45%). The compound is a mixture of rotamers. ^{1}H NMR (400 MHz, DMSO-d_6) δ 7.95 – 7.83 (m, 2.00H), 7.71 – 7.59 (m, 2.05H), 7.46 – 7.36 (m, 2.00H), 7.36 – 7.26 (m, 2.06H), 4.67 (ddd, J = 58.5, 10.9, 5.0 Hz, 0.79H), 4.51 – 4.40 (m, 0.54H), 4.38 – 4.30 (m, 0.53H), 4.30 – 4.21 (m, 0.98H), 3.91 – 3.77 (m, 1.06H), 3.54 – 3.44 (br s, 0.40H), 3.43 – 3.30 (m, 1.02H), 3.25 (d, J = 10.1 Hz, 0.44H), 3.15 (br s, 2.19H), 3.09 – 2.99 (m, 0.51H), 2.94 (br s, 1.19H), 2.35 – 2.21 (m, 0.56H); 2.13 – 1.99 (m, 0.42H), 1.94 – 1.47 (m, 4.08H), 1.02 (d, J = 7.0 Hz, 1.69H), 0.60 (d, J =

7.0 Hz, 1.22H); ^{13}C NMR (100 MHz, DMSO) δ 175.84, 153.75, 143.90, 140.80, 127.61, 127.05, 124.95, 120.10, 82.54, 81.45, 65.94, 65.09, 59.96, 58.39, 57.42, 47.02, 46.87, 46.35, 43.08, 24.70, 24.37, 23.95, 23.64, 13.97, 13.38. See Supplementary Fig. 12 for ^{1}H and ^{13}C NMR spectra. HRMS (ESI) calculated *m/z* for $C_{24}H_{28}NO_5$ $(M+H)^+$ 410.1967, found 410.1964.

2) Solid-phase synthesis of β-lactam MMAF

Reagents:

All reagents and resins were purchased from commercial sources unless otherwise referenced. Abbreviations: Fmoc-Dap-OH = Fmoc-dolaproline; Fmoc-Dil-OH = Fmoc-dolaisoleucine; TFA = trifluoroacetic acid; DIPEA = diisopropylethylamine; HATU = 1-[bis (dimethylamino) methylene]-1*H*-1, 2, 3-triazolo [4, 5-b]pyridinium 3-oxid hexafluoro-phosphate.

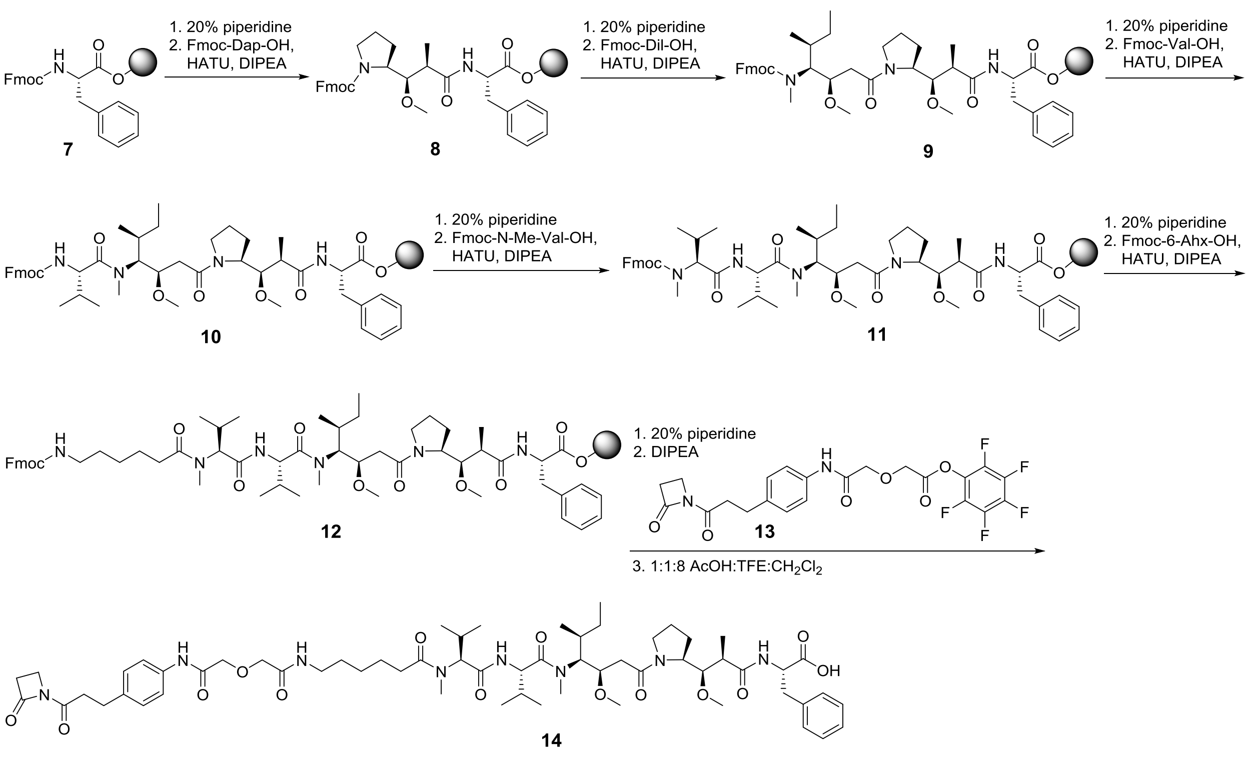

Solid phase synthesis:

Fmoc-Phe-OH (12 eq) dissolved in 4 mL of dry CH_2Cl_2 and 0.5 mL of DIPEA was added to 72 mg (0.80 meq/g) of pre-swollen 2-chlorotrityl resin. The mixture was agitated under argon (4 h), the solvent was removed, and the resin was washed [CH_2Cl_2 (x3), DMF (x3), and MeOH (x3)] to provide resin **7**. The resin was then deprotected with 20% piperidine in DMF (20 min, x2), washed with DMF (x3), and agitated with a freshly prepared solution of Fmoc-Dap-OH (3 eq) and HATU (3 eq) in 1.5 mL of DMF (4% (v/v) DIPEA) at rt (overnight). The solvent was removed and the resin was washed with DMF (x3) to provide resin **8**. The resin was then Fmoc-deprotected with 20% piperidine in DMF (20 min, x2), washed with DMF (x3), then agitated with a freshly prepared solution of Fmoc-Dil-OH (2.5 eq) and HATU (2.5 eq) in 2.5 mL of DMF (4% (v/v) DIPEA) at rt (7 h). The solvent was removed and the resin was washed with DMF (x3) to provide resin **9**. Unfunctionalized resin was capped with a solution of acetic anhydride (10:10:80 v/v Ac_2O:DIPEA:DMF) at rt (30 min). The solvent was removed, the resin was washed with DMF (x3), and Fmoc-deprotected with 20% piperidine in DMF (20 min, x2). The resin was washed with DMF (x3), then agitated with a freshly prepared solution of Fmoc-Val-OH (6.0 eq) and HATU (6.0 eq) in 4.2 mL of DMF (4% (v/v) DIPEA) at rt (overnight). The solvent was removed and the resin was washed with DMF (x3). Unfunctionalized resin was then capped with a solution of acetic anhydride (10:10:80 v/v Ac_2O:DIPEA:DMF) at rt (30 min) and washed with DMF (x3) to provide resin **10**. The mixture was Fmoc-deprotected with 20% piperidine in DMF (20 min, x2), washed with DMF (x3), and agitated with a freshly prepared solution of Fmoc-N-Me-Val-OH (5 eq) and HATU (5 eq) in 2.5 mL of DMF (4% (v/v) DIPEA) at rt (6 h) then washed with DMF (x3). Unfunctionalized resin was capped with a solution of acetic anhydride (10:10:80 v/v Ac_2O:DIPEA:DMF) at rt (30 min) and washed with DMF (x3) to

provide resin **11**. The mixture was Fmoc-deprotected with 20% piperidine in DMF (20 min, x2), washed with DMF (x3), then agitated with a freshly prepared solution of Fmoc-6-Ahx-OH (6 eq) and HATU (6 eq) in 2.5 mL of DMF (4% (v/v) DIPEA) overnight. The solvent was removed and the resin was washed with DMF (x3). Unfunctionalized resin was capped with a solution of acetic anhydride (10:10:80 v/v Ac_2O:DIPEA:DMF) at rt (30 min) and washed with DMF (x3) to provide resin **12**. The mixture was Fmoc-deprotected with 20% piperidine in DMF (20 min, x2), washed with DMF (x3), and agitated with a freshly prepared solution of β-lactam pentafluorophenyl (pfp) ester (**13**[3], 3 eq) in 3 mL of DMF (1% (v/v) DIPEA) for 1 h. The resin was washed, sequentially, with DMF and CH_2Cl_2. The resin was then treated with dilute AcOH solution (1:1:8 v/v AcOH:TFE:CH_2Cl_2; 2.5 mL x 30 min, x2) to cleave the peptide from the resin, and the resulting solution was concentrated *in vacuo*, and purified using reverse-phase HPLC to give final product **14** in high purity (see chromatogram below). Analytical HPLC trace was performed using an Agilent 1260 Infinity Quaternary pump with a Phenomenex Gemini C18 analytical column (250 x 4.6 mm, 5-µm particle size) and monitoring by UV absorbance at 215 nm. The chromatogram was obtained using the following gradient elution: 100% H_2O for 2 minutes, gradient of 100% H_2O to 100% CH_3CN over 28 min, and 100% CH_3CN for 2 min, followed by a column wash with 100% CH_3CN for 5 min and 3-min re-equilibration to 100% H_2O. HPLC mobile phase solvents contained 0.1% TFA. The compound is a mixture of rotamers. ^1H NMR (400 MHz, DMSO-d_6) δ 12.76 (br s, 0.82H), 9.97 (s, 0.97H), 8.56 (d, J = 8.7 Hz, 0.53H), 8.36 (dd, J = 8.8, 2.2 Hz, 0.50H), 8.19 – 8.05 (m, 1.49H) , 7.74 (d, J = 8.4 Hz, 0.24H), 7.64 (d, J = 8.6 Hz, 0.26H), 7.53 (d, J = 8.2 Hz, 2.0H), 7.30 – 7.12 (m, 7.35H), 6.99 (s, 0.24H), 5.58 (br s, 0.40H), 4.79 – 4.34 (m, 3.22H), 4.13 (s, 1.96H), 4.02 (s, 2.07H), 4.01 – 3.89 (m, 1.79H), 3.88 – 3.49 (m, 5.23H), 3.45 (t, J = 5.3 Hz, 2.93H), 3.36 – 3.26 (m, 1.31H), 3.24 (s,

1.35H), 3.23 – 2.99 (m, 13.04H), 2.96 (d, J = 8.0 Hz, 1.63H), 2.93 – 2.77 (m, 8.50H), 2.48 – 1.88 (m, 8.31H), 1.85 – 1.57 (m, 4.98H), 1.56 – 1.12 (m, 12.21H), 1.09 – 0.98 (m, 4.10H), 0.96 – 0.66 (m, 20.87H); ^{13}C NMR (175 MHz, DMSO) δ 173.42, 173.37, 173.24, 173.14, 172.92, 172.87, 172.63, 172.59, 172.35, 169.83, 169.73, 169.65, 169.39, 168.94, 168.71, 168.67, 167.69, 167.67, 165.75, 157.96, 157.78, 156.61, 137.72, 137.52, 137.48, 136.32, 135.94, 128.92, 128.68, 128.64, 128.50, 128.05, 128.03, 126.28, 126.21, 119.99, 85.34, 81.36, 77.52, 70.73, 70.61, 64.20, 64.18, 60.93, 60.85, 60.75, 60.18, 58.50, 58.15, 57.11, 57.07, 55.77, 55.09, 54.03, 53.89, 52.99, 51.89, 51.81, 47.50, 47.08, 46.13, 43.08, 42.89, 41.36, 38.16, 38.09, 37.42, 37.15, 36.41, 36.35, 35.56, 35.08, 33.35, 32.77, 32.56, 32.52, 31.92, 31.57, 31.28, 30.52, 30.20, 30.01, 29.94, 29.08, 29.00, 28.75, 28.50, 28.47, 27.54, 26.26, 26.21, 26.19, 26.08, 25.32, 25.26, 25.18, 24.97, 24.94, 24.46, 24.36, 24.27, 23.10, 19.19, 19.15, 18.97, 18.94, 18.91, 18.77, 18.75, 18.70, 18.67, 18.48, 18.42, 18.31, 18.10, 15.63, 15.54, 15.40, 15.33, 15.18, 14.86, 11.03, 10.41, 10.22. See Supplementary Fig. 13 for ^{1}H and ^{13}C NMR spectra. HRMS (ESI) calculated m/z for $C_{61}H_{93}N_8O_{14}$ $(M+H)^+$ 1161.6811, found 1161.6780. Chromatogram of purified β-lactam MMAF, **14**:

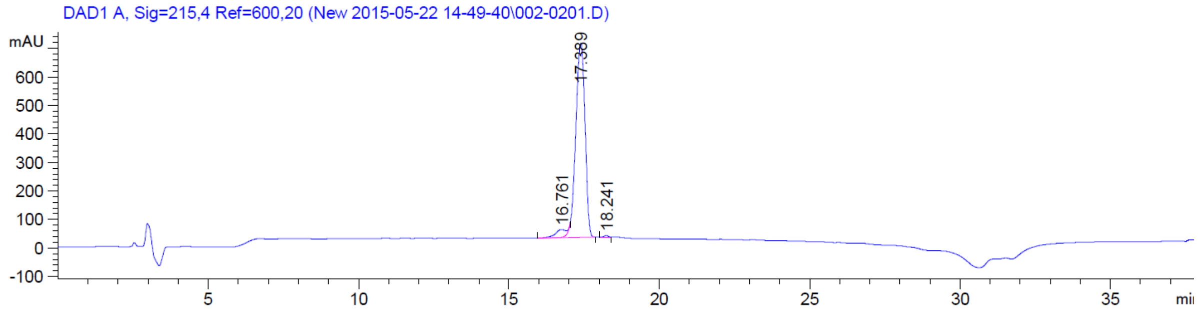

Supplementary References

1. Nelson, C.G. & Burke, T.R. Samarium iodide-mediated Reformatsky reactions for the stereoselective preparation of beta-hydroxy-gamma-amino acids: synthesis of isostatine and dolaisoleucine. *J. Org. Chem.* **77**, 733-738 (2012).
2. Shioiri, T., Hayashi, K. & Hamada, Y. Stereoselective synthesis of dolastatin 10 and its congeners. *Tetrahedron* **49**, 1913-1924 (1993).
3. Magano, J. *et al.* Chromatography- and lyophilization-free synthesis of a peptide-linker conjugate. *Organic Process Research & Development* **18**, 142-151 (2014).
